# The sequence alignment problem: boundary conditions as the unifying principle

**DOI:** 10.1093/bib/bbag333

**Published:** 2026-06-21

**Authors:** Paul A Gagniuc, Elvira Gagniuc

**Affiliations:** Faculty of Engineering in Foreign Languages, National University of Science and Technology Politehnica Bucharest, Department of Engineering in Foreign Languages, 313 Splaiul Independenței, 5th District, Bucharest RO-060042, Romania; Faculty of Veterinary Medicine, University of Agronomic Sciences and Veterinary Medicine, Department of Paraclinical Sciences, 105 Splaiul Independenței, 5th District, Bucharest RO-050097, Romania

**Keywords:** sequence alignment, global alignment, local alignment, dynamic programing, affine gap model, substitution matrix, Smith–Waterman, Needleman–Wunsch, scoring theory, algorithm optimization, GPU acceleration, FPGA accelerator, seed-extend method, sparse DP, genomic analysis, protein domain analysis, variant discovery, metagenomic analysis, privacy-preserving computation

## Abstract

Sequence alignment provides a formal framework for comparison of biological sequences through score maximization over matches, mismatches, and insertion–deletion events. Classical formulations distinguish between global alignment, which enforces end-to-end correspondence through fixed boundary conditions, and local alignment, which extracts high-scoring subsequences without global consistency. Both paradigms arise from the same dynamic programing (DP) recurrences, shaped by substitution matrices and gap-penalty models that approximate molecular evolution. Canonical algorithms such as Needleman–Wunsch and Smith–Waterman establish the foundations of exact alignment, while later extensions introduce affine and convex gap costs, statistical score distributions, and probabilistic significance models. Modern work builds on these principles through bit-parallel techniques, band-restricted computation, cache-aware layouts, single instruction, multiple data and graphics processing unit parallelism, hardware accelerators, and index-assisted heuristics that enable large-scale genomic analysis. Sequence alignment underpins applications ranging from whole-genome comparison and metagenomics to protein annotation, variant detection, human leukocyte antigen typing, and microbial surveillance. Persistent challenges include scalability to ultra-long sequences, faithful models of complex mutation processes, avoidance of parameter bias, and formal limits on exact subquadratic solutions. Emerging directions emphasize adaptive data-driven scoring, hybrid global–local formulations, privacy-preserving computation, and real-time or incremental alignment. These developments reaffirm sequence alignment as a closely related DP framework shaped primarily by boundary conditions rather than distinct paradigms.

## Introduction

The sequence alignment problem concerns the systematic comparison of two biological sequences in order to identify an arrangement of residues that maximizes a defined similarity score under a set of constraints imposed by an objective function. This optimization task can be expressed formally as the search for a pair of strings derived from the original sequences by the insertion of gap characters. The resulting alignment maximizes a scoring function based on residue matches, mismatches, and indels. This formulation was introduced in its global form by Needleman and Wunsch [1] and later extended in more specialized frameworks [2]. The motivation for a rigorous distinction between global and local alignment lies in the fact that homologous full-length sequences should be aligned over their entire span to capture conserved architecture. In contrast, sequences that diverge substantially or contain domain rearrangements require an algorithm that isolates high scoring subsequences without the need for a full-length correspondence. This principle was formalized in the Smith–Waterman local alignment method [3]. These scoring models rely on substitution matrices that encode the empirical likelihood of residue replacement derived from evolutionary observations, such as point accepted mutation (PAM) and BLOcks SUbstitution Matrix (BLOSUM) families [4]. They also rely on gap cost functions that penalize insertion and deletion events according to linear, affine, or convex schemes that approximate the biological reality of indel formation [5]. These components collectively define an objective function whose maximization determines the optimal alignment under the assumed evolutionary or structural model. Variations in these functions have produced a substantial diversity of algorithmic refinements across decades of research [6]. The present review surveys the foundational algorithms that established global and local alignment as core computational primitives in molecular biology, examines subsequent improvements in dynamic programing (DP), scoring design, and boundary conditions, and contrasts the theoretical properties that distinguish the two paradigms in both formal and practical terms [1, 3, 7]. The literature discussed in this review was selected on the basis of direct relevance to sequence alignment theory, methodological contribution, and conceptual significance. Particular emphasis was placed on boundary conditions, scoring models, computational complexity, and modern optimization strategies. A schematic overview of this literature-selection procedure is provided as Supplementary Fig. S1.

**Unifying viewpoint.** In this review, we treat global, local, semi-global, and “glocal” alignment as boundary-condition realizations of one DP optimization problem. The recurrence defines a score surface over the same lattice, while boundary initialization, admissible start/stop states, and termination rules determine which paths represent valid solutions. This framing places “global” and “local” on a continuum of boundary constraints rather than as separate paradigms, which becomes essential for understanding modern hybrid formulations that combine anchored global coherence with locally optimal segments. While this work emphasizes boundary conditions as a primary structural mechanism, alignment behavior also depends on scoring models, gap penalties, and statistical assumptions, which jointly define the solution space.

It also synthesizes modern developments such as bit-parallel acceleration, single instruction, multiple data (SIMD) and graphics processing unit (GPU)-based optimizations, memory-efficient formulations, and application-specific enhancements that enable alignment at scale in contemporary genomics [8]. We also update the discussion of state-of-the-art exact and long-read alignment techniques and connect them to the same boundary-conditioned formulation. Moreover, this work constitutes a structured narrative review with thematic synthesis and outlines open problems that continue to motivate research, namely—alignment under complex evolutionary models, efficient handling of ultra-long sequences, and the integration of learning-based scoring functions into classical DP frameworks [9].

## Mathematical and algorithmic foundations

Sequence alignment is defined as the optimization of a scoring function under explicit boundary conditions within a DP framework. The formulation below establishes the notation and constraints used in subsequent analysis. Global and local sequence alignment rest on a shared DP framework but differ in problem constraints, boundary conditions, and optimization objectives. Formal problem statements expose these differences at a mathematical level, while scoring models and gap-penalty schemes determine the structure of admissible solutions. Computational complexity further delineates the feasibility of exact alignment across sequence lengths and application domains.

### Formal problem statements

Global sequence alignment can be expressed as the optimization of an objective function over two complete sequences $X={x}_1{x}_2\dots{x}_m$ and $Y={y}_1{y}_2\dots{y}_n$. The task is to identify augmented strings ${X}^{\prime }$ and ${Y}^{\prime }$ produced by the insertion of gap characters such that the alignment score $S\left({X}^{\prime },{Y}^{\prime}\right)$ is maximized under a predefined scoring model. This formulation stems from the classical DP framework in which the optimal alignment is defined as the highest scoring path across a 2D lattice whose boundaries are fully constrained to enforce end-to-end comparison [10]. Local alignment, in contrast, seeks the maximum-scoring pair of subsequences rather than entire sequences. Mathematically, this requires the identification of subsequences ${X}_{i:j}$ and ${Y}_{k:\ell }$ that maximize $S\left({X}_{i:j},{Y}_{k:\ell}\right)$ without enforcement of alignment from sequence termini. This principle was codified in the definition of the maximum local similarity problem [11]. The DP recurrences reflect these conceptual differences: in global alignment, the score matrix *F* satisfies:


$$F\left(i,j\right)=\max \left\{\begin{array}{@{}l}F\left(i-1,j-1\right)+s\left({x}_i,{y}_j\right),\\{}F\left(i-1,j\right)-g,\\{}F\left(i,j-1\right)-g\end{array}\right.{\displaystyle \begin{array}{cc}& \\{}& \\{}& \end{array}}$$

with boundary initialization $F\left(0,j\right)=- gj$ and $F\left(i,0\right)=- gi$, whereas local alignment introduces the additional condition:


$$F\left(i,j\right)=\max \left\{\begin{array}{@{}l}0,\\{}F\left(i-1,j-1\right)+s\left({x}_i,{y}_j\right),\\{}\begin{array}{@{}c}F\left(i-1,j\right)-g,\\{}F\left(i,j-1\right)-g\end{array}\end{array}\right.{\displaystyle \begin{array}{cc}& \\{}& \\{}& \\{}& \end{array}}$$

which allows the recurrence to restart whenever negative contributions accumulate, which produces an unconstrained interior optimum [11, 12]. These differences in boundary conditions are central to the algorithmic and biological interpretation of each method.

### Scoring models

The scoring functions that underpin these optimization processes rely on substitution matrices that reflect empirical models of amino acid or nucleotide replacement over evolutionary timescales. PAM matrices are derived from Markovian models of accepted point mutations [13]. BLOSUM matrices are constructed from conserved blocks of related sequences [14]. Both encode residue specific log odds scores that guide alignment decisions across the DP lattice. Gap penalties are incorporated to account for insertion and deletion events and usually take one of three forms. Linear penalties assign a fixed cost to each indel. Affine penalties of the form $g(k)={g}_o+k\bullet{g}_e$ for *k≥ 1* assign a higher cost to gap opening than to gap extension, where ${g}_o$ denotes the gap-opening penalty and ${g}_e$ the gap-extension penalty. Convex models seek to approximate biologically realistic length dependent indel formation [15]. The choice of penalty model has important theoretical implications. Linear penalties may produce fragmented alignments, whereas affine penalties create more coherent indel blocks and reduce combinatorial branching in optimal paths. Convex penalties are more expressive, but they often require modified recurrences or heuristic approximations to maintain tractable computation [16]. These models influence not only alignment plausibility but also the shape of the DP landscape, the density of competitive paths, and the statistical properties of optimal scores.

### Computational complexity

The standard DP algorithms for global and local alignment operate in *O(mn)* time for sequences of lengths *m* and *n*. Each state in the alignment matrix is computed from a constant number of predecessor states. This complexity bound has remained inescapable for exact alignment under classical scoring models [17]. The space complexity is likewise $O\left(m\bullet n\right)$ in its naïve form because the full matrix must be retained to allow traceback. The divide and conquer method introduced by Myers and Miller reduces the required memory to $O\left(m+n\right)$ while it preserves optimality through a recursive decomposition of the alignment path [7, 12]. These theoretical characteristics ensure that both global and local alignment remain optimally solvable under DP. The monotonicity and optimal substructure properties embedded in the recurrences guarantee correctness of the solutions produced by the algorithms. Although numerous heuristics, sparse DP techniques, and index-guided extensions have been proposed to accelerate practical alignment, the canonical algorithms remain the gold standard against which approximate or heuristic methods are evaluated [18]. The preceding formulation defines the common DP structure and boundary assumptions that underlie the alignment variants examined in the following sections.

## Classical global alignment algorithms

Global alignment enforces end-to-end sequence correspondence under DP constraints. This section reviews the Needleman–Wunsch framework and key extensions, including gap models, memory optimization, boundary variants, and statistical behavior.

### Needleman–Wunsch framework

The classical global alignment algorithm introduced by Needleman and Wunsch constructs an *m× n* DP matrix *F* in which each entry represents the optimal alignment score for the prefixes ${X}_{1:i}$ and ${Y}_{1:j}$, and the recurrence:


$$F\left(i,j\right)=\max \left\{F\left(i-1,j-1\right)+s\left({x}_i,{y}_j\right),F\left(i-1,j\right)-g,F\left(i,j-1\right)-g\right\}$$

operates under boundary conditions $F\left(0,j\right)=- gj$ and $F\left(i,0\right)=- gi$, which ensure that the alignment spans both sequences in their entirety [1, 19]. The correctness of this framework follows from its optimal substructure and monotonicity properties. Each optimal global alignment path can be decomposed into optimal subpaths defined over successive prefixes. This fact guarantees the global optimality of the final score under any additive scoring scheme [20]. The DP formulation remains the canonical method for end-to-end alignment because it provides exact solutions and admits rigorous proofs of optimality under general scoring models.

### Gap models and the Gotoh improvement

The introduction of affine gap penalties transformed the computational landscape of global alignment by enabling biologically meaningful distinctions between gap opening and extension events:


$$\mathrm{gap}(k)={g}_o+k{g}_e,$$

with ${g}_o$ the opening penalty and ${g}_e$ the extension penalty, can be computed efficiently by the use of three coupled matrices *M*, ${I}_x$, and ${I}_y$ that encode match/mismatch states and gap states in each direction [2, 21]. These recurrences:


\begin{align*} M\left(i,j\right)& =\max\kern-1pt \left\{M\left(i-1,j-1\right)\kern-2pt,{I}_x\left(i-1,j-1\right)\kern-2pt,{I}_y\left(i-1,j-1\right)\right\}\kern-1pt+s\left({x}_i,{y}_j\right)\kern-2pt,\\{}{I}_x\left(i,j\right)& =\max\kern-1pt \left\{M\left(i-1,j\right)-{g}_o,{I}_x\left(i-1,j\right)-{g}_e\right\}\kern-1pt,\\{}{I}_y\left(i,j\right)& =\max\kern-1pt \left\{M\left(i,j-1\right)-{g}_o,{I}_y\left(i,j-1\right)-{g}_e\right\} \end{align*}

achieve the same asymptotic time complexity as the linear-gap Needleman–Wunsch algorithm while they enables gap structures that better reflect evolutionary mechanisms, especially in long sequences where indel blocks are common [22]. In practical applications, affine scoring improves alignment stability, reduces spurious gap fragmentation, and increases biological realism in protein and DNA analyzes.

### Algorithmic variants of global alignment

Several structural modifications extend global alignment to specialized contexts. Semi-global or overlap alignments modify the boundary conditions so that terminal gaps are either unpenalized or selectively scored. This formulation is useful in settings such as adapter trimming or fragment alignment, where only internal regions should contribute to the final score [23]. For sequences that are nearly identical but differ by a small edit distance, the DP matrix can be restricted to a diagonal band of width *k*. This yields a reduced problem whose computation scales as $O\left(k\cdotp \max \left\{m,n\right\}\right)$. The method has proven valuable in long-read alignment and error-tolerant matching [24]. Linear-space global alignment, derived from the Myers–Miller divide-and-conquer method, reconstructs optimal alignments without storing the full DP matrix [7]. It exploits the fact that the alignment path can be partitioned into two subpaths that meet at a midpoint computed through forward and reverse DP scans [7, 12]. These structural variants preserve optimality while substantially reducing resource requirements in large-scale genomic analyzes.

### Statistical behavior of global alignment

The distribution of optimal global alignment scores under random sequence models has played an important role in understanding the sensitivity and specificity of global methods. Early probabilistic studies demonstrated that, under independent and identically distributed sequence models, alignment scores obey asymptotic behavior that can be approximated through large-deviation principles. This result gives insight into the expected background score landscape and the minimal score necessary to infer homology [25]. In practice, global alignment deteriorates when sequences undergo domain rearrangements, circular permutations, or recombination events that disrupt collinearity. Such processes violate the implicit assumption that homologous residues appear in order along both sequences [19, 26]. Models of structural shuffling and recombination further show that global end-to-end comparison becomes misleading in the presence of mosaic architectures, gene fusions, or mobile domain insertions. In such contexts, local or graph-based alignment strategies provide more accurate representations of evolutionary relationships [19, 27].

## Classical local alignment algorithms

Local alignment detects high-scoring subsequences without end-to-end constraints and is suited to cases where similarity occurs only over restricted regions. This section presents the Smith–Waterman formulation, affine-gap extensions, and the statistical interpretation of local scores.

### Smith–Waterman framework

**Figure 1:**
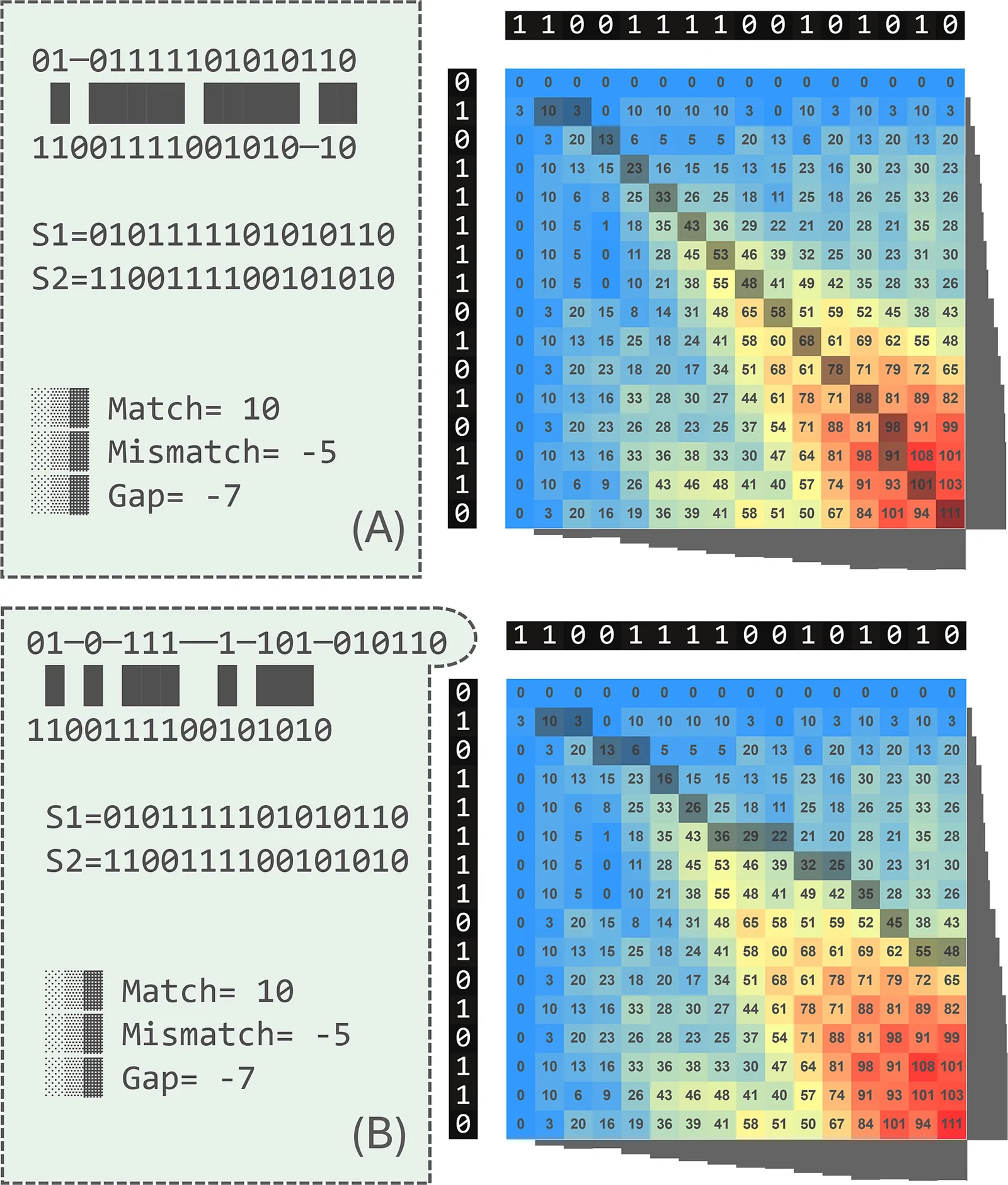
Local alignment score surface with core detection and bidirectional sequence reconstruction; the scoring scheme is match = +10, mismatch = −5, and gap penalty = −7; the boundary condition follows the Smith–Waterman formulation, with a zero lower bound; (A) DP score matrix for local alignment; the maximum score marks, the endpoint of the optimal local alignment core, and the canonical Smith–Waterman solution; the color gradient represents score magnitude; (B) the same score matrix with an alternative internal traceback origin that does not coincide with the global maximum; traceback follows the same recurrence rules but extends beyond the local core through explicit prefix and suffix reconstruction, illustrating a controlled transition from strict local alignment toward hybrid local–global behavior.

Local alignment identifies the highest-scoring subsequences within two strings through a modification of the DP formulation of global alignment so that negative partial scores cannot propagate (Fig. 1 and Supplementary File S1). The Smith–Waterman algorithm introduces the zero-reset rule into the recurrence:


$$H\left(i,j\right)\!=\!\max \left\{0,H\left(i-1,j-1\right)\!+\!s\left({x}_i,{y}_j\right),H\left(i-1,j\right)\!-\!g,H\left(i,j-1\right)\!-\!g\right\},$$

where $H\left(i,j\right)$ denotes the local score, $s\left({x}_i,{y}_j\right)$ denotes the substitution score, and *g>0* denotes the gap penalty. The zero-reset term discards any unfavorable prefix and allows the alignment to start at any matrix position $\left(i,j\right)$ with a positive score [3, 19, 28]. The traceback process begins at the global maximum of the matrix *H* rather than at its boundary and proceeds backward until the score first returns to zero, which yields the optimal local alignment with no requirement for end-to-end correspondence [29]. This mechanism captures conserved motifs and domains even when the surrounding regions have diverged beyond alignability. This property makes the method essential for the detection of functionally relevant similarity within heterogeneous sequences. Complete pseudocode for both algorithms is provided in Appendix A.

### Affine-gap local alignment

The extension of affine gap penalties to local alignment adopts the Gotoh formulation through the use of three linked matrices *M*, ${I}_x$, and ${I}_y$, with preservation of the zero-reset condition that terminates low-scoring paths. In this formulation, the match matrix follows:


$$M\left(i,j\right)=\max \left\{M\left(i-1,j-1\right),{I}_x\left(i-1,j-1\right),{I}_y\left(i-1,j-1\right)\right\}+s\left({x}_i,{y}_j\right),$$

and each gap matrix satisfies:


$${I}_x\left(i,j\right)=\max \left\{M\left(i-1,j\right)-{g}_o,{I}_x\left(i-1,j\right)-{g}_e\right\},$$

$${I}_y\left(i,j\right)=\max \left\{M\left(i,j-1\right)-{g}_o,{I}_y\left(i,j-1\right)-{g}_e\right\},$$

while the final score at $\left(i,j\right)$ is defined as $H\left(i,j\right)=\max \left\{0,M\left(i,j\right),\right. \left.{I}_x\left(i,j\right),{I}_y\left(i,j\right)\right\}$ to preserve locality [19, 21, 30]. This affine-gap local DP scheme substantially improves the sensitivity of the algorithm to indels by alignment of gap structures that better reflect evolutionary processes. As a result, it increases the likelihood of recovery of biologically meaningful motifs in protein or nucleotide sequences. [31].

### Statistical significance

The statistical characterization of local alignment scores is grounded in the Karlin–Altschul theory. This theory establishes that the distribution of maximal local alignment scores under an independent and identically distributed null model converges to an extreme value distribution of the Gumbel type. In this framework, the probability that the optimal score *S* exceeds a given threshold is approximated by:


$$P\left(S\ge x\right)\approx 1-\exp \left(- Kmn\;{e}^{-\lambda x}\right),$$

where *x* is the alignment score threshold, while *m* and *n* are the lengths of the two compared sequences. *K* accounts for the effective number of possible local alignments under the random background model, and *λ* accounts for the scoring system (i.e. match scores, mismatch penalties, gap penalties, and background residue frequencies) used to compute the alignment score *S* [32]. This result directly motivates the computation of *E*-values, defined as the expected number of alignments with score at least *x* that occur by chance in a random database. These values form the statistical backbone of widely used local alignment tools such as Basic Local Alignment Search Tool (BLAST) [33]. The analytical dependence of these parameters on substitution matrices and gap penalties highlights the deep connection between algorithmic design and probabilistic interpretation in local alignment. The question that arises is: When is local alignment superior? Local alignment excels when sequences contain conserved motifs embedded within divergent or rearranged backgrounds. Under these conditions, global methods fail because collinearity is absent across the entire alignment span. Motif discovery pipelines frequently rely on local alignment to reveal short conserved sequence patterns that encode binding sites or catalytic residues, particularly in protein families where functional motifs remain stable despite extensive sequence drift [34].

At the domain level, local alignment is indispensable for the identification of homologous structural modules that may appear in different contexts due to recombination, exon shuffling, or gene fusion. These phenomena disrupt global synteny but preserve localized similarity [35]. In evolutionary or population-level analyzes, local alignment further proves to be essential for correct detection of recombination hotspots and mosaic architectures [36]. It is also essential for the alignment of short metagenomic fragments whose limited length and heterogeneous origin preclude meaningful global comparison [36].

## Algorithmic comparison: global versus local

**Figure 2:**
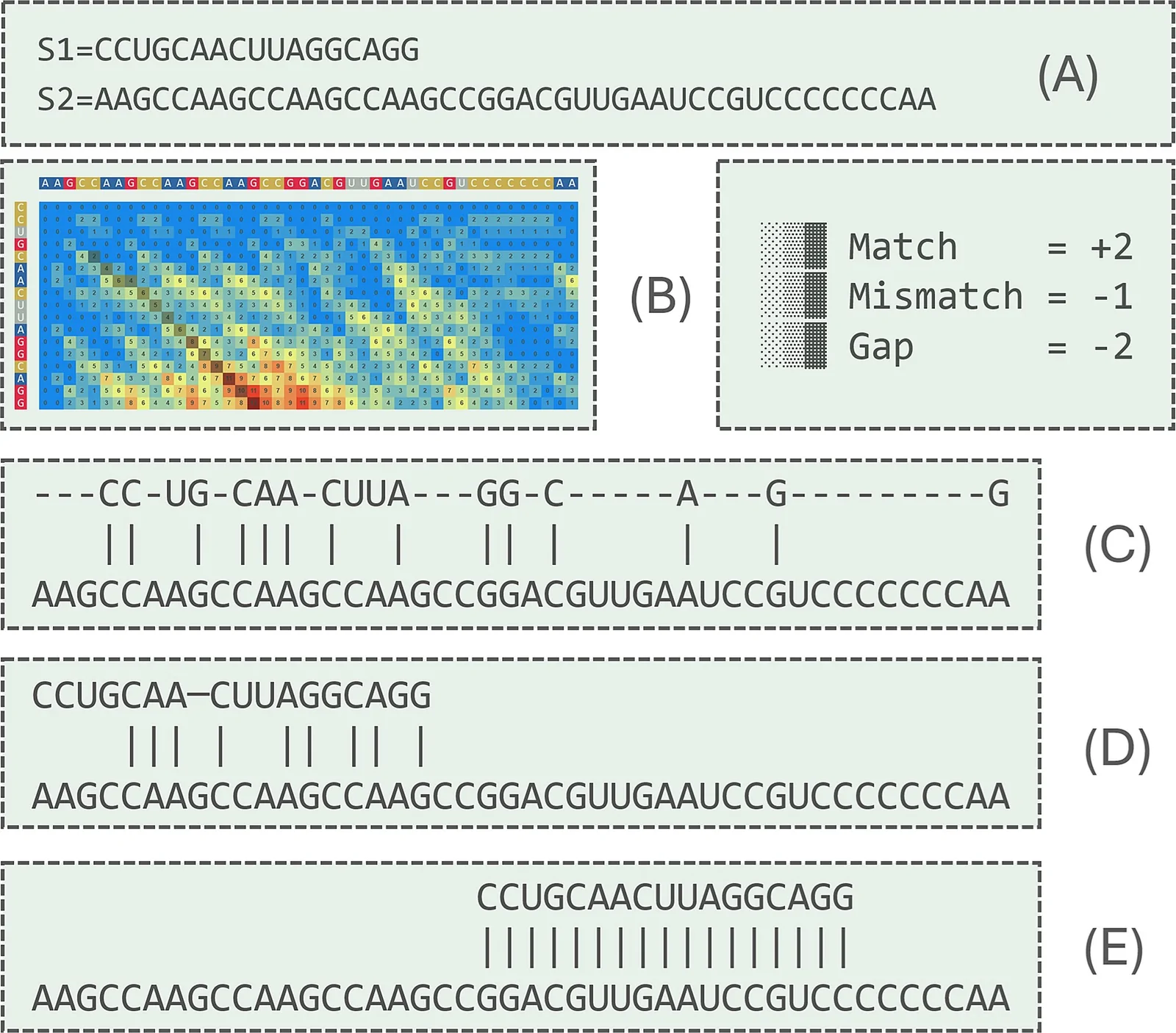
Comparative illustration of global and local alignment outcomes under a unified scoring scheme; the scoring scheme uses match = +2, mismatch = −1, and gap penalty = −2; global alignment uses fixed end-to-end boundary conditions, whereas local alignment allows internal start and stop positions; (A) input nucleotide sequences S1 and S2; (B) local DP score matrix, with color intensity denoting score magnitude; (C) global alignment under fixed boundary constraints, with gaps used to preserve full sequence correspondence; (D) local alignment extracted from the highest-scoring internal region; (E) local alignment under complementarity constraints, showing a continuous region of strong correspondence within the aligned core.

This comparative analysis treats global and local alignment as two boundary-condition realizations of the same DP optimization problem. Their practical differences arise primarily from initialization, admissible start/stop states, and termination rules, which reshape the set of valid paths on an otherwise shared score surface (Table 1). A direct comparison clarifies how these boundary choices influence sensitivity, specificity, robustness to divergence, and interpretation across biological contexts (Fig. 2 and Supplementary File S1). The following analysis contrasts global and local alignment at the level of recurrences, score behavior, and empirical use cases in order to delineate their respective domains of applicability.

**Table 1:** Technical comparison of global and local alignment.

Property	Global alignment	Local alignment
Boundary conditions	Fixed sequence ends	Free internal start and stop
Initialization	Cumulative terminal gap penalties	Zero initialized boundaries
Termination	Bottom right cell	Highest scoring cell
Traceback	From bottom right to origin	From maximum score to zero
Terminal gaps	Penalized	Not scored outside local core
Main objective	Aligns full sequences	Aligns best subsequences
Statistical interpretation	Full length score	Extreme value local score
Best suited for	Similar, collinear sequences	Motifs, domains, fragments
Main limitation	Sensitive to rearrangement	Loses full sequence context

The two formulations share the same DP framework but differ in boundary initialization, termination, traceback, terminal gap treatment, and score interpretation.

### Boundary conditions and recurrence differences

The contrast between global and local alignment emerges most clearly in the structure of their DP tables, where distinct boundary conditions induce fundamentally different optimality landscapes. In the global formulation, the initialization $F\left(0,j\right)=- gj$ and $F\left(i,0\right)=- gi$ forces the alignment path to traverse the entire matrix from the upper-left to the lower-right corner, which allows for an uninterrupted trajectory that reflects end-to-end correspondence between sequences [37]. In local alignment, the introduction of the zero-reset condition $H\left(i,j\right)=\max \left\{0,\dots \right\}$ yields a matrix in which isolated high-scoring regions appear as basins surrounded by zero-valued borders. This structure produces a landscape with multiple admissible maxima rather than a single constrained path [28]. The discrepancy between these recurrence structures implies that global alignment constructs a deterministic optimal path across the full domain, whereas local alignment discovers an embedded optimum whose location and extent depend entirely on the overall scoring dynamics [38]. Modern aligners rarely instantiate purely “global” or purely “local” behavior. Many pipelines adopt hybrid boundary choices: they enforce global coherence over a chained set of anchors, while they apply local or glocal DP inside the anchor corridors or at breakpoints. In this view, seed-and-extend, chaining, overlap alignment, and semi-global alignment represent practical mechanisms that continuously interpolate between strict end-to-end constraints and fully unconstrained local optima. These observations support the interpretation of global and local alignment as special cases of a unified boundary-conditioned optimization framework.

### Sensitivity versus specificity trade-offs

Global and local alignment also differ in their intrinsic sensitivity and specificity properties. Because global alignment enforces complete sequence coverage, its scoring is stable when conservation is relatively uniform across the entire length of both sequences. Such stability enables a robust discrimination between homologs and non-homologs in cases where evolutionary divergence is moderate and structural order is preserved [39]. Local alignment, by contrast, is tuned to detect short high-scoring islands within otherwise unrelated or noisy regions. As a result, it is significantly more sensitive to conserved motifs, catalytic residues, or short functional elements that persist despite extensive divergence [34]. This difference means that the local algorithm may detect biologically meaningful relationships even when large portions of the sequences share no detectable similarity. In contrast, the global algorithm may dilute such localized evidence under a large background of mismatches or gaps [40].

### Behavior under noisy or divergent conditions

The behavior of global and local alignment under high noise or structural divergence depends critically on the choice of scoring matrices and gap penalties. Substitution matrices with high selectivity amplify the contrast between conserved and non-conserved positions. This effect accentuates the strengths of local alignment in motif-rich environments but may degrade the performance of global alignment when divergence is unevenly distributed [41]. Gap models exert similar influence. In the global setting, affine penalties with large opening costs tend to favor long contiguous alignments, which can mask biologically relevant recombination or rearrangement events [22]. However, in the local setting, the same penalties suppress excessive fragmentation that might otherwise arise from noisy or low complexity sequences [22]. Each algorithm therefore exhibits characteristic biases: global alignment favors collinear homology and penalizes interruptions aggressively, whereas local alignment preferentially highlights compact regions of similarity and ignores global structural context. Consequently, this distinction directly affects the interpretation of alignments in evolutionary and functional analyzes [42].

### Practical comparison on synthetic and real examples

Practical evaluation of the two algorithms on synthetic and empirical datasets demonstrates circumstances in which each method excels or fails. In controlled synthetic benchmarks where sequences differ primarily by point mutations and uniform indels, global alignment consistently recovers the intended correspondence with high fidelity. On the other hand, local alignment may truncate alignments prematurely due to boundary zeros that interrupt continuity [43]. Real biological sequences often exhibit more complex behavior—proteins composed of modular domains or sequences shaped by recombination events are more accurately compared by using local alignment, which isolates high-confidence segments even when global collinearity is violated [35]. In metagenomic workflows, short reads derived from heterogeneous species mixtures typically align more reliably under local criteria because only a small fraction of each fragment may correspond to a homologous region in any given database entry [36]. These examples illustrate that the choice between global and local alignment is intrinsically task-dependent, governed by biological expectations about sequence organization, conservation patterns, and the evolutionary processes that shape the underlying data [44]. From a comparative perspective, modern alignment variants differ primarily in their boundary assumptions, scoring models, and computational trade-offs. Methods that enforce strict global boundary constraints provide consistent end-to-end agreement but may lose sensitivity under structural divergence, whereas local and semi-global formulations relax boundary conditions to capture partial similarity at the cost of reduced global coherence. Hybrid and adaptive strategies interpolate between these extremes through flexible boundary selection, anchor constraints, or adaptive scoring schemes. These differences reflect distinct assumptions about sequence relatedness, error models, and computational efficiency, which determine the suitability of each method for specific biological contexts. Furthermore, modern approaches extend classical formulations through relaxed boundary constraints, adaptive scoring, or heuristic acceleration, which improve sensitivity and scalability but may introduce approximation error or increased model complexity. Computational feasibility also varies substantially across alignment strategies. Namely, classical DP methods provide exact optimal solutions but require quadratic time and memory, which limits scalability for long sequences or large datasets. Heuristic and seed-based approaches reduce computational cost through approximate search and indexing strategies, which improve scalability at the cost of potential sensitivity loss. Parallel implementations and hardware acceleration further enhance performance but also introduce additional architectural constraints and implementation complexity.

## Computational optimizations and modern variants

Sequence alignment remains grounded in DP, yet practical use at genomic scale demands algorithmic reformulation beyond the canonical *O(mn)* paradigm. Methods in this area fall into four categories: exact, heuristic, approximate, and conditionally exact methods. Exact methods compute optimal solutions under the defined scoring model through full DP evaluation. Heuristic methods, such as seed and extend strategies, lower computational cost through search space reduction and may miss the optimal solution. Approximate methods accept controlled deviation from the optimal solution to handle larger datasets. Conditionally exact methods still return the optimal solution, but only when the true alignment path remains inside the restricted search region, such as a fixed band around the main diagonal or a predefined edit distance limit. Advances in computer architecture, memory hierarchy, and parallel hardware have motivated a broad class of optimizations that preserve alignment optimality while they reduce time or space cost under realistic assumptions about sequence similarity. These developments range from bit-level reformulation of recurrence evaluation to architectural adaptation on modern central processing units (CPUs), GPUs, and dedicated hardware. Additional efficiency emerges through heuristic restriction of the search space via indexing, anchoring, and sparsity exploitation, which limits DP evaluation to regions of high relevance. The variants reviewed in this section illustrate how classical alignment theory adapts to contemporary computational constraints without an abandonment of its formal foundations.

### Bit-parallel and band-limited variants

Bit-parallelism provides one of the most influential accelerations of exact sequence alignment because it replaces explicit DP cell updates with bitwise operations executed across entire machine words. Myers introduced a bit-vector formulation in order to compute edit distances, in which the state of each DP column is compactly represented by bit masks, thereby enabling an update cost of $O\left(\left\lceil m/w\right\rceil \right)$ per column for a word size *w*, while it preserves exact optimality under unit-cost edit models [45]. Although originally developed for Levenshtein distance, the bit-vector principle extends to global and local alignment variants through modified recurrence encodings that approximate affine penalties or incorporate band constraints. Band-limited DP further reduces computational load, as it restricts the computation to cells that satisfy $\mid i-j\mid \le k$, with time complexity $O\left(k\cdotp \max \left\{m,n\right\}\right)$, a strategy that retains optimality when sequence divergence is sufficiently small that the alignment path must remain close to the diagonal [46]. Together, bit-parallel and band-limited methods introduce asymptotic and practical gains that are especially advantageous for long or repetitive sequences. Recent work on exact alignment optimization includes the wavefront alignment (WFA) algorithm, which reformulates DP evaluation by tracking wavefronts of increasing edit distance rather than filling the entire matrix [47]. This approach reduces computational cost for long and highly similar sequences and preserves exact optimality. The WFA algorithm represents a state-of-the-art method for exact sequence alignment and shows how algorithmic reformulation supports architectural acceleration strategies [47].

### Cache-aware, single instruction, multiple data, and graphics processing unit optimizations

Modern microarchitectures permit additional acceleration of alignment algorithms through cache-aware memory tiling, SIMD vectorization, and GPU parallelization. Cache-aware DP partitions the DP matrix into blocks optimized for locality, which yields substantial performance gains through a reduction of cache misses during traversal of large matrices [48]. SIMD instruction sets such as Advanced Vector Extensions (AVX)2 and AVX-512 enable parallel evaluation of multiple DP cells per instruction, and optimized implementations of Smith–Waterman have exploited these operations to achieve multi-fold speedups on contemporary CPUs [49]. GPU-based parallelization builds on wavefront evaluation of the DP matrix, which allows for a computation of anti-diagonals concurrently while it maintains dependency correctness. Such GPU implementations have yielded high-throughput local alignment engines capable of processing billions of residues per second on modern devices [50]. These improvements reposition classical DP methods as competitive tools in large-scale genomic pipelines where high-throughput sequence comparison is required.

### Hardware accelerators

Field-programmable gate arrays (FPGA) and application-specific integrated circuits (ASICs) have long been explored as platforms for acceleration of sequence alignment, particularly for Smith–Waterman, due to their capacity to implement deep pipelines of parallel cell updates. FPGA-based architectures achieve fixed-latency, high-throughput evaluation of DP recurrences by mapping each cell computation to a physical processing element in a systolic array, which enables near-real-time performance for moderate-sized matrices [51]. ASIC implementations push these advantages further and integrate specialized logic for substitution scoring, gap handling, and affine-penalty evaluation. Therefore, ASIC systems trade flexibility for maximal throughput in industrial or clinical workflows where speed outweighs configurability [52]. These hardware accelerators highlight an inherent trade-off between accuracy and speed: while they compute exact alignments, their fixed memory and architecture constraints may limit applicability to extremely long or dynamically parameterized alignment tasks.

### Index-guided alignment (seed-and-extend)

Heuristic alignment methods improve efficiency, as they combine indexing with localized DP. Tools such as BLAST employ a seed-and-extend strategy in which short exact or near-exact matches are first identified through hash or trie indices, after which local DP is applied only in windows that surround these seeds [33]. More recent mappers, such as Burrows-Wheeler Aligner (BWA) and Minimap2, build on compressed Ferragina Manzini (FM) index structures and minimizer sketches to locate approximate seed positions efficiently. Seed extension then proceeds through either full local DP or glocal DP, which aligns one sequence end-to-end while it allows internal boundaries on the other [18, 53]. The distinction between global and local extension is critical: global extension is used when reads are expected to match entire reference segments, whereas local extension enables alignment of fragments, structural variants, or rearranged domains that would be truncated under strict end-to-end criteria.

### Sparse dynamic programing, anchoring, and heuristic filters

Sparse DP reduces computation through a restriction of the DP evaluation to candidate regions identified by anchor points or high-confidence matches. Anchoring selects a set of collinear seed matches and computes an optimal chain under a scoring function that maximizes global consistency, while it excludes implausible regions from full DP evaluation [54]. Sparse DP then fills only the corridors between adjacent anchors, often using pruning rules that eliminate DP states whose partial scores fall below bounds derived from expected edit distances or statistical constraints [55]. These techniques achieve large speedups in practical alignment tasks and explore the fact that biological sequences often contain long stretches of predictable collinearity punctuated by relatively few structural variations.

## Applications and choice over global and local alignment

The practical choice between global and local alignment depends less on algorithmic preference than on biological context, data structure, and analytical objective. Sequence length, fragmentation, evolutionary distance, and structural variability all influence whether end-to-end comparison or localized similarity provides the most reliable interpretation. Application domains impose distinct constraints that favor one alignment paradigm over the other, or require controlled combinations of both. The next subsections examine how this choice manifests across major areas of modern computational biology, from genome-scale comparison to clinical diagnostics, and illustrate how alignment strategy adapts to domain-specific data properties and biological expectations.

### Genomics

In comparative genomics, the choice between global and local alignment depends on the expected structural correspondence between genomes and the evolutionary processes that shape their architecture. Whole-genome alignment typically applies global or semi-global strategies to reconstruct syntenic blocks across large chromosomal regions [56]. Such strategies allow investigators to infer rearrangements, detect homologous loci, and assess conservation on a macro-evolutionary scale [56]. When genomes remain largely collinear, global alignment recovers ordered homologous segments with high reliability, whereas local alignment becomes essential in regions where structural variation has disrupted global organization through inversions, translocations, or segmental duplications [57]. Homolog identification across large phylogenetic distances benefits from localized scoring models because conserved coding segments or regulatory motifs may persist even when flanking sequences have diverged extensively through lineage specific events [58].

### Transcriptomics and metagenomics

In transcriptomic analysis, reads that originate from RNA sequencing experiments often contain splicing junctions, sequence biases, and technology-specific noise sources that make local alignment strategies necessary. Aligners designed for RNA-seq employ variants of local and “glocal” DP to reconcile intron–exon boundaries without the need for full-length matching. This requirement is central to accurate isoform quantification and transcript reconstruction [59]. Metagenomic samples introduce even greater heterogeneity: short fragments from multiple species coexist in the same sample, which makes global alignment infeasible because each read may contain only a small region homologous to any given database sequence. Local alignment therefore underpins taxonomic classification, functional annotation, and abundance estimation in microbiome studies which support partial-match detection despite substantial compositional complexity and uneven coverage [36]. These applications highlight how statistical properties of local scoring adapt naturally to noisy or fragmentary contexts encountered in high-throughput sequencing.

### Structural and functional bioinformatics

Protein structural and functional annotation relies heavily on local alignment because protein evolution frequently proceeds through the reshuffling, duplication, and recombination of modular domains. Domain-level comparison benefits from local scoring models that isolate strongly conserved structural modules even when their placement within the full-length sequence has changed through architectural rearrangements [35]. Protein family classification and motif discovery likewise exploit local alignment to detect conserved catalytic residues, binding signatures, and recurrent sequence motifs that remain stable despite shifts in global fold or topology [42]. This principle is widely used in profile- and hidden Markov model-based classification pipelines [42]. The capacity of local alignment to highlight short but functionally significant subsequences accounts for its central role in structural bioinformatics and homology-based annotation methods.

### Medical and clinical applications

Medical genomics depends on the precise alignment of sequencing data for variant discovery, where global and local strategies coexist as a function of the properties of the data. Germline variant detection pipelines often apply global or semi-global alignment when reads are long and exhibit limited structural divergence from the reference genome. Such conditions enable accurate base-level comparison and high-confidence identification of single-nucleotide polymorphisms and indels [60]. In contrast, somatic mutation analysis and structural variant calling require local alignment around breakpoints. In these regions, rearrangements, duplications, or translocations disrupt global correspondence and necessitate scoring systems that accommodate locally high similarity embedded in structurally complex contexts [61]. Clinical applications such as human leukocyte antigen typing demand extremely fine-grained discrimination between closely related alleles. In this setting, local alignment captures the highly polymorphic exonic regions that distinguish haplotypes, while it ignores intronic or regulatory sequences of limited diagnostic relevance [62]. Microbial surveillance further relies on local alignment to match pathogen signatures from clinical or environmental samples, where rapid detection depends on the identification of short diagnostic motifs in the presence of sequencing noise and strain diversity [63].

## Limitations, challenges, and open research questions

Despite decades of refinement, sequence alignment remains constrained by fundamental computational, statistical, and model limits. Classical DP provides exact solutions under simplified assumptions, yet modern biological data increasingly violate these assumptions through scale, structural complexity, and heterogeneous mutation processes. As sequence length, data volume, and application sensitivity continue to increase, both algorithmic design and theoretical analysis expose tensions between optimality, realism, and feasibility. This section examines the principal limitations of current alignment paradigms, identifies unresolved challenges that arise at the intersection of biology and computation, and outlines research directions that seek to extend alignment theory beyond its traditional boundaries.

### Support for ultra-long sequences

The growth of sequencing technologies has produced ultra-long reads and chromosome-scale assemblies whose alignment challenges the classical *O(mn)* DP formulation. Memory use becomes prohibitive when matrices cannot be streamed or partitioned effectively, and time requirements escalate beyond practical limits in population-scale comparative analyzes [64]. Approximate alignment schemes that guarantee bounded deviation from the optimal score address these constraints through exploitation of sparsity induced by long exact matches. A second route uses metric embeddings for dimensionality reduction with preservation of alignment-relevant structures [65]. Such methods provide theoretical assurances of correctness within a known error margin, yet their performance depends strongly on the distribution of similarity across the sequences being compared, which in turn reveals an unresolved tension between scalability and provable optimality.

### Complex mutation models

Biological sequences often evolve under mutation processes that deviate substantially from the assumptions encoded in classic affine gap penalties. Non-affine costs that model superlinear gap opening, context-dependent indels, or microsatellite expansion require recurrence relations more flexible than the Gotoh framework, but naïve DP implementations of such models often expand computational complexity to impractical levels [66]. Structural rearrangements, which include translocations, inversions, duplications, and domain shuffling, break the global collinearity assumption inherent in the DP lattice. This in fact renders classical global alignment misleading and necessitates graph-based or segmented local approaches to capture mosaic evolutionary histories [19, 67, 68]. A graph-based representation replaces the single linear reference with a labeled graph whose alternative paths encode population variation, repeated segments, and rearranged blocks. Alignment then becomes the search for a high-scoring walk through the graph that best explains the query sequence, which generalizes classical DP from a rectangular lattice to a graph-structured state space. In this setting, “boundary conditions” correspond to the choice of admissible entry and exit nodes (or subgraphs), the permitted path topology, and the constraints imposed on traversal, rather than to sequence termini alone. This perspective is central in the pangenomics era, where inversions, translocations, and complex structural variation violate linear collinearity. Graph topology allows an alignment path to accommodate non-collinear events without a misleading end-to-end correspondence along a single coordinate system. As a result, global coherence is enforced at the level of graph traversal, while locality is expressed through boundary choices that restrict traversal to a region of the graph or allow free start/stop within it. We therefore view sequence-to-graph mapping as an extension of the same boundary-conditioned optimization principle, where the reference structure changes from a line to a graph. Moreover, these phenomena highlight the partial inadequacy of simple scoring functions to capture the complexity of molecular evolution and justify the search for alignment models that better capture real mutation processes at feasible computational cost.

### Algorithmic bias in scoring models and theoretical limits

The quality of an alignment depends sensitively on the scoring matrix and gap parameters used to evaluate residue pairings. Substitution matrices derived from biased training sets or inappropriate evolutionary models can introduce systematic misalignment through overemphasis on substitutions characteristic of specific protein families or through underestimation of the frequency of others [69]. Optimal parameterization remains an open problem: although empirical tuning approaches such as maximum-likelihood fitting over curated alignments can refine matrices and gap costs, the lack of universally accepted ground truth for large protein families limits the reproducibility and generalizability of these methods [70]. This uncertainty translates into algorithmic bias, where apparently optimal alignments reflect artifacts of the scoring system rather than biological reality, which reinforces the need for models that adaptively infer scoring parameters from data. Theoretical analysis reveals fundamental limits on the efficiency of exact alignment algorithms. Under common complexity assumptions, no subquadratic-time exact algorithm exists for global or local alignment with arbitrary scoring schemes because the standard DP formulation reduces to the longest common subsequence problem. For this reason, conditional lower bounds imply that strongly subquadratic solutions would violate widely believed conjectures in fine-grained complexity theory [71]. More general alignment formulations, which include those that incorporate context-sensitive gap costs or position-specific substitution models, can be shown to be nondeterministic polynomial-time (NP)-hard. This makes polynomial-time exact solutions unlikely unless structural constraints or approximations are imposed [72]. These hardness results delineate the boundaries of algorithmic innovation and clarify why heuristic, probabilistic, or approximate schemes remain central to modern high-performance alignment pipelines.

### Future directions

Emerging research directions attempt to reconcile biological realism with computational feasibility. Learning-based scoring functions, such as deep neural models that infer substitution preferences or context-dependent gap patterns, offer the possibility of replacement for hand-crafted scoring matrices with data-driven models that adapt to protein families or taxonomic groups [73]. Hybrid frameworks that integrate global and local information reflect a common strategy for handling structural rearrangements [74]. Such frameworks stitch together localized DP results within a global graphical scaffold and preserve coherence of large-scale comparisons. Secure or “privacy-preserving” alignment methods, which use homomorphic encryption or secure multiparty computation, enable sensitive genomic data to be aligned without direct exposure, a requirement in clinical genomics and cross-institutional studies [75]. Incremental or streaming alignment algorithms aim to align data as it is produced by sequencers. This leads to a reduction in memory overhead and preserves near-real-time responsiveness for diagnostic and environmental surveillance applications [76]. Thus, these directions indicate a shift toward adaptive, hybrid, and privacy-aware alignment frameworks that preserve the rigor of classical DP while they extend its applicability to future genomic scales and societal constraints. All these advanced developments build on the established practical impact of sequence alignment methods in contemporary genomic analysis. For example, large-scale genome sequencing and variant detection rely on accurate read alignment to identify mutations and structural variation [77, 78]. In infectious disease surveillance, alignment-based methods enable rapid analysis of viral genomes and tracking of evolutionary changes [79]. Similarly, comparative genomics and transcriptome analysis depend on alignment techniques to identify conserved regions and functional elements [80]. These applications illustrate the central role of alignment algorithms in modern biological research and data-driven medicine. Recent advances also include foundation models in computational biology and multimodal learning frameworks that integrate sequence, structural, and functional information within unified representations [81, 82]. Related developments in deep learning architectures, including attention-based models and hierarchical neural representations, demonstrate the broader impact of data-driven approaches across biomedical and computational domains [83–85]. These novel approaches enable context-aware alignment scoring and representation learning across large biological datasets, which extend classical sequence comparison beyond hand-crafted similarity measures [86]. Such advances indicate a shift toward data-driven alignment frameworks that combine statistical learning with DP principles.

## Conclusion

Global and local sequence alignment remain foundational constructs in computational biology, and their complementary strengths continue to shape modern analytical workflows across genomics, transcriptomics, structural bioinformatics, and clinical applications. Global alignment provides a rigorous framework for end-to-end comparison through the enforcement of continuity across entire sequences, an approach that excels when structural collinearity and uniform evolutionary conservation are preserved. Local alignment, by contrast, isolates high-scoring regions embedded within divergent or rearranged contexts, which in turn enables the detection of motifs, domains, and functional elements that persist despite extensive evolutionary remodeling. The mathematical differences between these paradigms are encoded primarily in their boundary conditions, recurrence relations, and optimality landscapes. These differences translate directly into their biological and computational behavior, which dictates when each strategy is appropriate and how each should be interpreted.

Advances in algorithmic optimization, hardware acceleration, and index-guided heuristics have extended the practical reach of classical DP methods. As a result, exact or near-exact alignment can be performed at scales unimaginable at the time of their original formulation. At the same time, contemporary genomic datasets expose the limitations of traditional models, especially in the presence of ultra-long sequences, complex mutation processes, and large structural rearrangements. These challenges motivate new theoretical and practical directions, among which we include learning-based scoring schemes, hybrid global–local frameworks, secure alignment protocols, and streaming algorithms capable of processing data in real time. The long-lasting relevance of global and local alignment lies not only in their historical importance but also in their adaptability. As sequencing technologies continue to evolve and biological questions grow in complexity, alignment algorithms must integrate richer evolutionary models, scalable computational techniques, and context-aware decision frameworks. The synthesis presented in this review underscores the essential role of alignment in modern computational biology and highlights the ongoing need for algorithmic innovation that balances mathematical rigor, biological interpretability, and computational efficiency.

**Key Points**

- Global and local sequence alignment form related dynamic programing (DP) formulations whose apparent distinction arises primarily from boundary conditions.
- Classical algorithms such as Needleman–Wunsch and Smith–Waterman differ in optimality landscapes, statistical interpretation, and biological scope, yet share the same mathematical foundation.
- Scoring models, gap penalties, and boundary constraints jointly determine alignment behavior, sensitivity, and robustness under evolutionary divergence and structural rearrangement.
- Modern optimizations including bit parallel methods, single instruction, multiple data execution, graphics processing unit acceleration, hardware pipelines, and sparse DP extend exact alignment to genomic-scale data without sacrificing theoretical rigor.
- Persistent challenges include extremely long sequences, complex mutation processes, scoring bias, and conditional lower bounds that preclude exact subquadratic solutions.

## Supplementary Material

Supplementary_material_bbag333

## Data Availability

No new data were generated or analyzed in this study. The visualization material, implementation examples, and supplementary resources used to generate the figures are provided in the Supplementary Material.
