# Supplementary material for: The sequence alignment problem: boundary conditions as the unifying principle
Source: Brief Bioinform. 2026 Jun 21;27(3):bbag333. doi: 10.1093/bib/bbag333 (PMC13283437; doi:10.1093/bib/bbag333)
Supplement: Supplementary_material_bbag333 [file supplementary_material_bbag333.zip › appendix_bbag333.docx]

**Appendix**

*A.1. Global Pairwise Alignment*

*Legend for Global Pairwise Alignment Algorithm.* This algorithm defines the canonical dynamic programming formulation for end-to-end sequence comparison. The matrix $F$stores optimal prefix scores under a fixed substitution score and constant gap cost. Boundary initialization enforces full coverage of both sequences and guarantees that any valid solution spans from the first to the last symbol. Each internal cell evaluates three transitions that correspond to match or mismatch, deletion, or insertion, and selects the maximum value to preserve optimal substructure. The traceback step reconstructs the alignment by retracing score-consistent transitions from the terminal cell $\left( m , n \right)$ to the origin, which yields a globally optimal correspondence and its associated score.

| Algorithm 1 → Needleman-Wunsch global DP |
| --- |
| Algorithm GlobalAlignment(X, Y, Score, GapCost)  Input:  X = x₁…xₘ  Y = y₁…yₙ  Score(a, b): substitution score  GapCost < 0  Output:  global alignment of X and Y  optimal global score  Create matrix F of size (m+1) × (n+1)  F[0,0] ← 0  For i from 1 to m  F[i,0] ← F[i−1,0] + GapCost  End For  For j from 1 to n  F[0,j] ← F[0,j−1] + GapCost  End For  For i from 1 to m  For j from 1 to n  diag ← F[i−1,j−1] + Score(xᵢ, yⱼ)  up ← F[i−1,j] + GapCost  left ← F[i,j−1] + GapCost  F[i,j] ← max(diag, up, left)  End For  End For  Set i ← m, j ← n  A ← empty string  B ← empty string  While i > 0 or j > 0  If i > 0 and j > 0 and  F[i,j] = F[i−1,j−1] + Score(xᵢ, yⱼ)  Prepend xᵢ to A  Prepend yⱼ to B  i ← i−1, j ← j−1  Else If i > 0 and F[i,j] = F[i−1,j] + GapCost  Prepend xᵢ to A  Prepend '-' to B  i ← i−1  Else  Prepend '-' to A  Prepend yⱼ to B  j ← j−1  End If  End While  Return A, B, F[m,n]  End Algorithm |

*A.2. Local Pairwise Alignment*

*Legend for Local Pairwise Alignment Algorithm.* This algorithm describes the classical local alignment formulation that detects high-score subsequences within larger sequence contexts. The matrix $H$ applies the same recurrence structure as the global case but introduces a zero lower bound that suppresses propagation of negative partial scores. The algorithm tracks the maximal value in the matrix and its coordinates, which define the endpoint of the optimal local region. Traceback begins at this maximal cell and terminates upon encounter of a zero-valued state, which isolates the highest-score subsequence pair. This formulation favors compact regions of strong similarity and excludes unrelated flanking segments by design.

| Algorithm 2 → Smith-Waterman local DP |
| --- |
| Algorithm LocalAlignment(X, Y, Score, GapCost)  Input:  X = x₁…xₘ  Y = y₁…yₙ  Score(a, b): substitution score  GapCost < 0  Output:  local alignment of X and Y  optimal local score  Create matrix H of size (m+1) × (n+1)  For all i, j  H[i,j] ← 0  End For  bestScore ← 0  bestI ← 0  bestJ ← 0  For i from 1 to m  For j from 1 to n  diag ← H[i−1,j−1] + Score(xᵢ, yⱼ)  up ← H[i−1,j] + GapCost  left ← H[i,j−1] + GapCost  H[i,j] ← max(0, diag, up, left)  If H[i,j] > bestScore  bestScore ← H[i,j]  bestI ← i  bestJ ← j  End If  End For  End For  Set i ← bestI, j ← bestJ  A ← empty string  B ← empty string  While i > 0 and j > 0 and H[i,j] > 0  If H[i,j] = H[i−1,j−1] + Score(xᵢ, yⱼ)  Prepend xᵢ to A  Prepend yⱼ to B  i ← i−1, j ← j−1  Else If H[i,j] = H[i−1,j] + GapCost  Prepend xᵢ to A  Prepend '-' to B  i ← i−1  Else  Prepend '-' to A  Prepend yⱼ to B  j ← j−1  End If  End While  Return A, B, bestScore  End Algorithm |
