# Supplementary material for: The sequence alignment problem: boundary conditions as the unifying principle
Source: Brief Bioinform. 2026 Jun 21;27(3):bbag333. doi: 10.1093/bib/bbag333 (PMC13283437; doi:10.1093/bib/bbag333)

## Slide 1
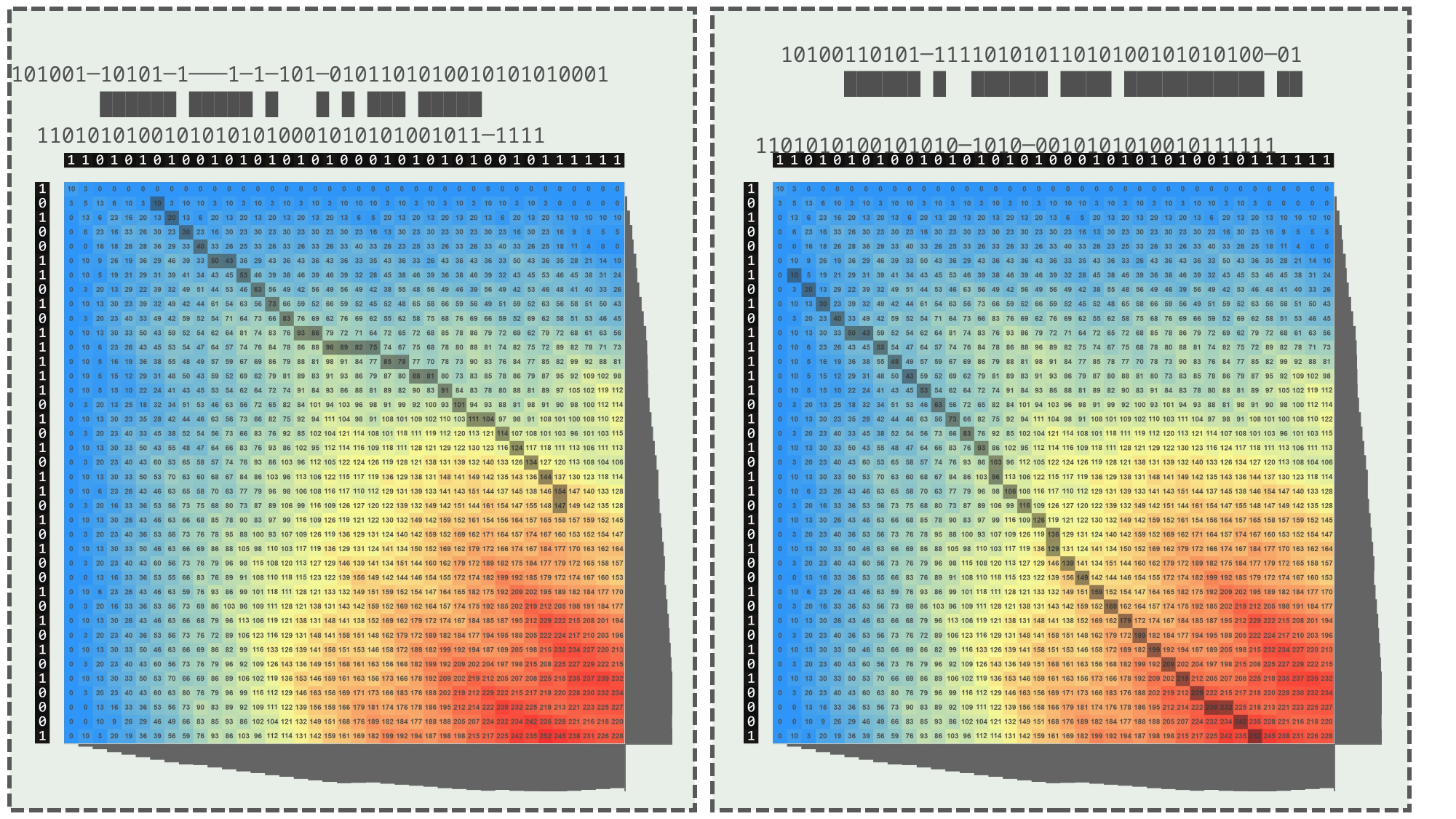

101001─10101─1───1─1─101─0101101010010101010001  
       ██████ █████ █   █ █ ███ █████ 
  11010101001010101010001010101001011─1111
  10100110101─11110101011010100101010100─01
       ██████ █  ██████ ████ ███████████ ██
       1101010100101010─1010─0010101010010111111

## Slide 2
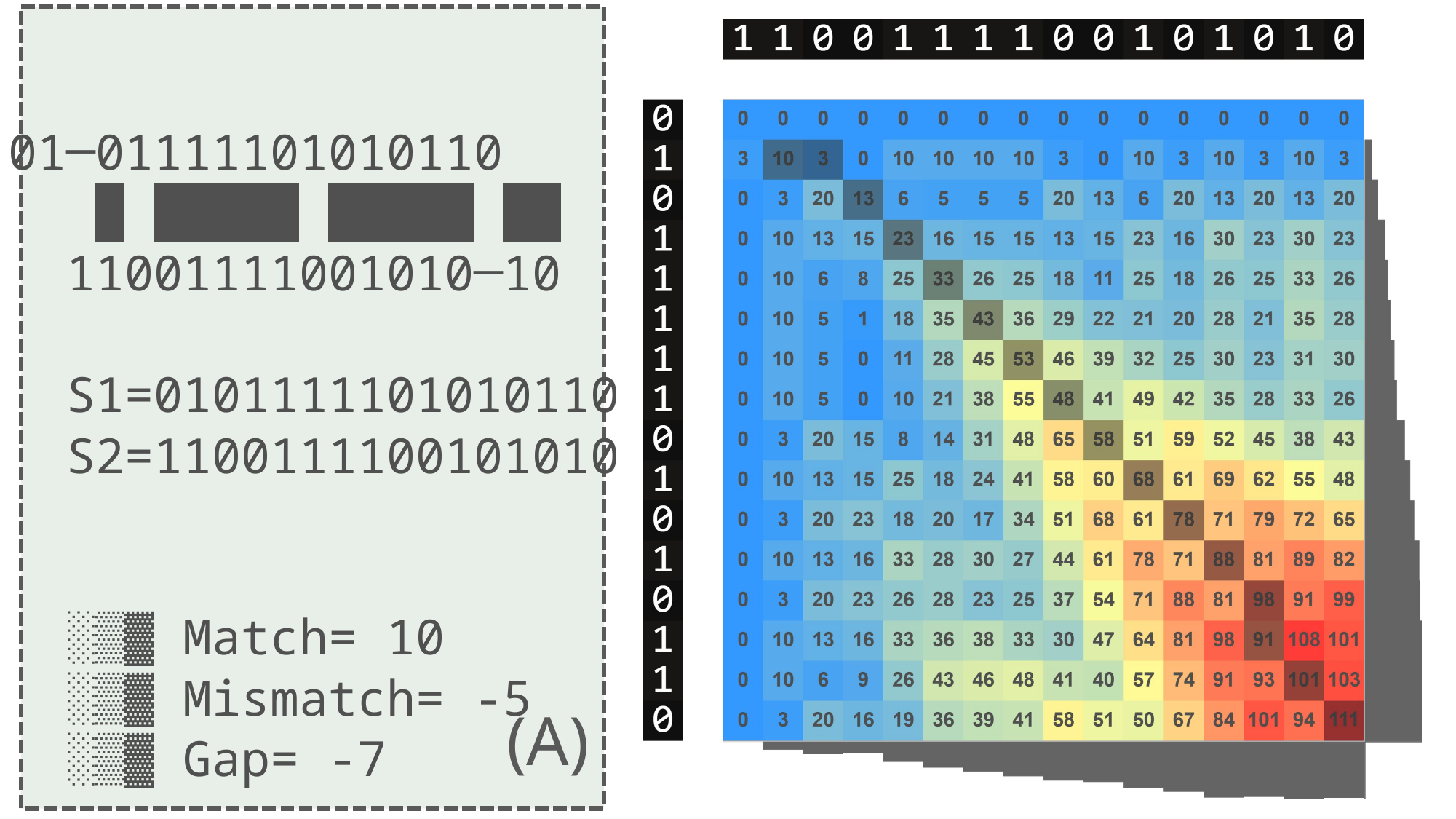

01─01111101010110
   █ █████ █████ ██
  11001111001010─10
 S1=0101111101010110
 S2=1100111100101010
 ░▒▓ Match= 10
 ░▒▓ Mismatch= -5
 ░▒▓ Gap= -7
(A)

## Slide 3
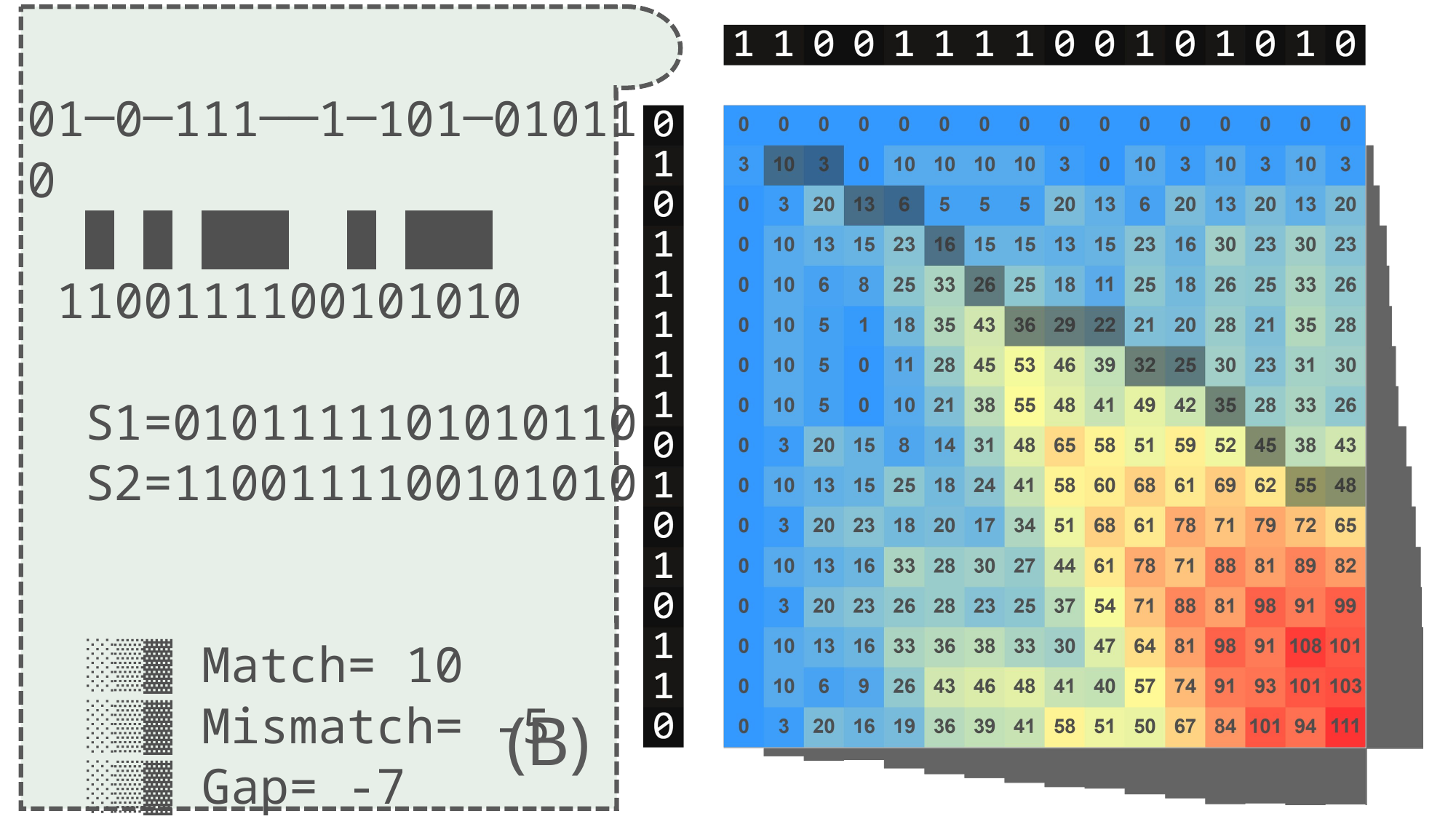

01─0─111──1─101─010110
  █ █ ███  █ ███
 1100111100101010
 S1=0101111101010110
 S2=1100111100101010
 ░▒▓ Match= 10
 ░▒▓ Mismatch= -5
 ░▒▓ Gap= -7
(B)

## Slide 4
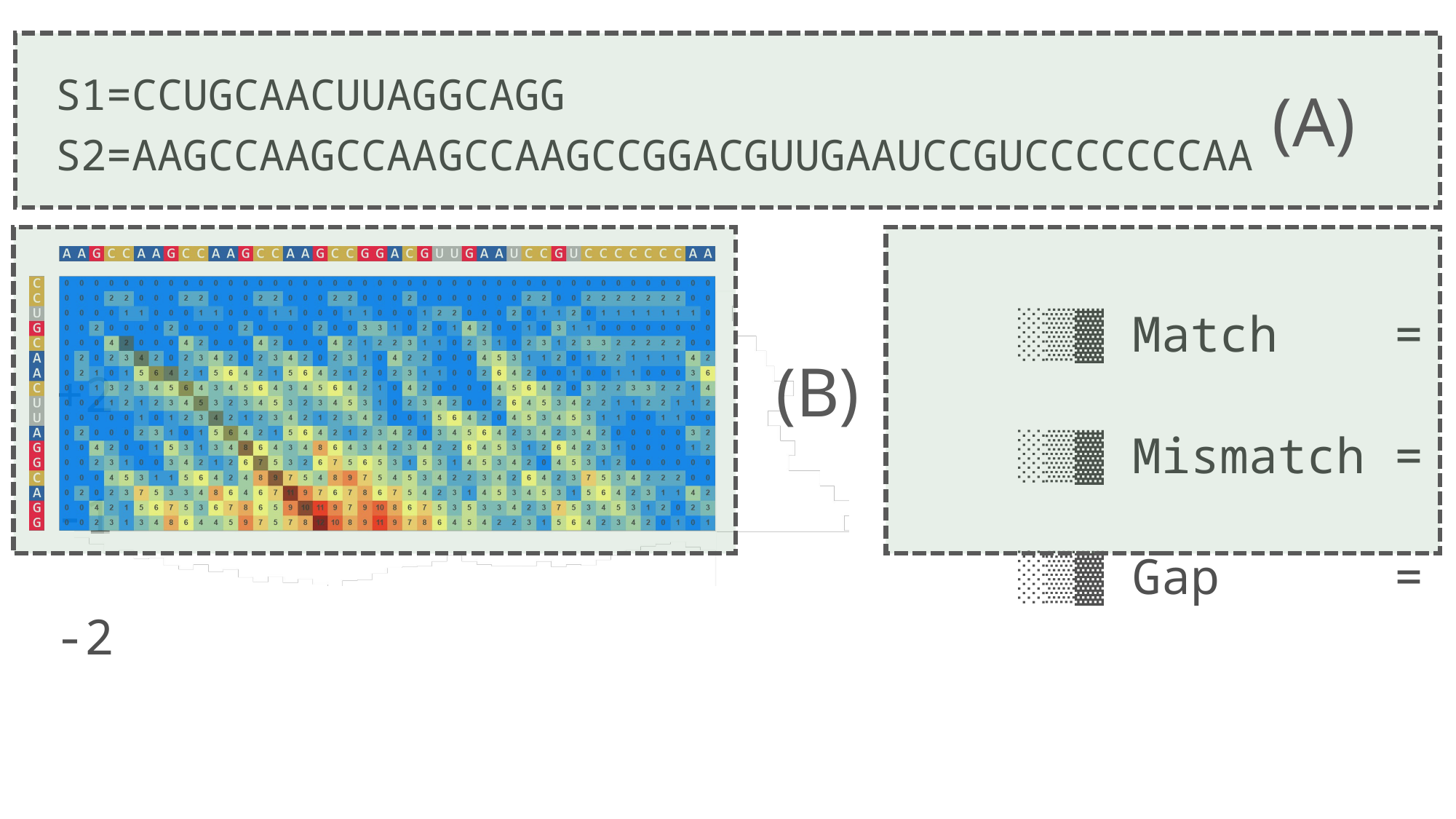

S1=CCUGCAACUUAGGCAGG
S2=AAGCCAAGCCAAGCCAAGCCGGACGUUGAAUCCGUCCCCCCCAA
 ░▒▓ Match = +2
 ░▒▓ Mismatch = -1
 ░▒▓ Gap = -2
(A)
(B)

## Slide 5
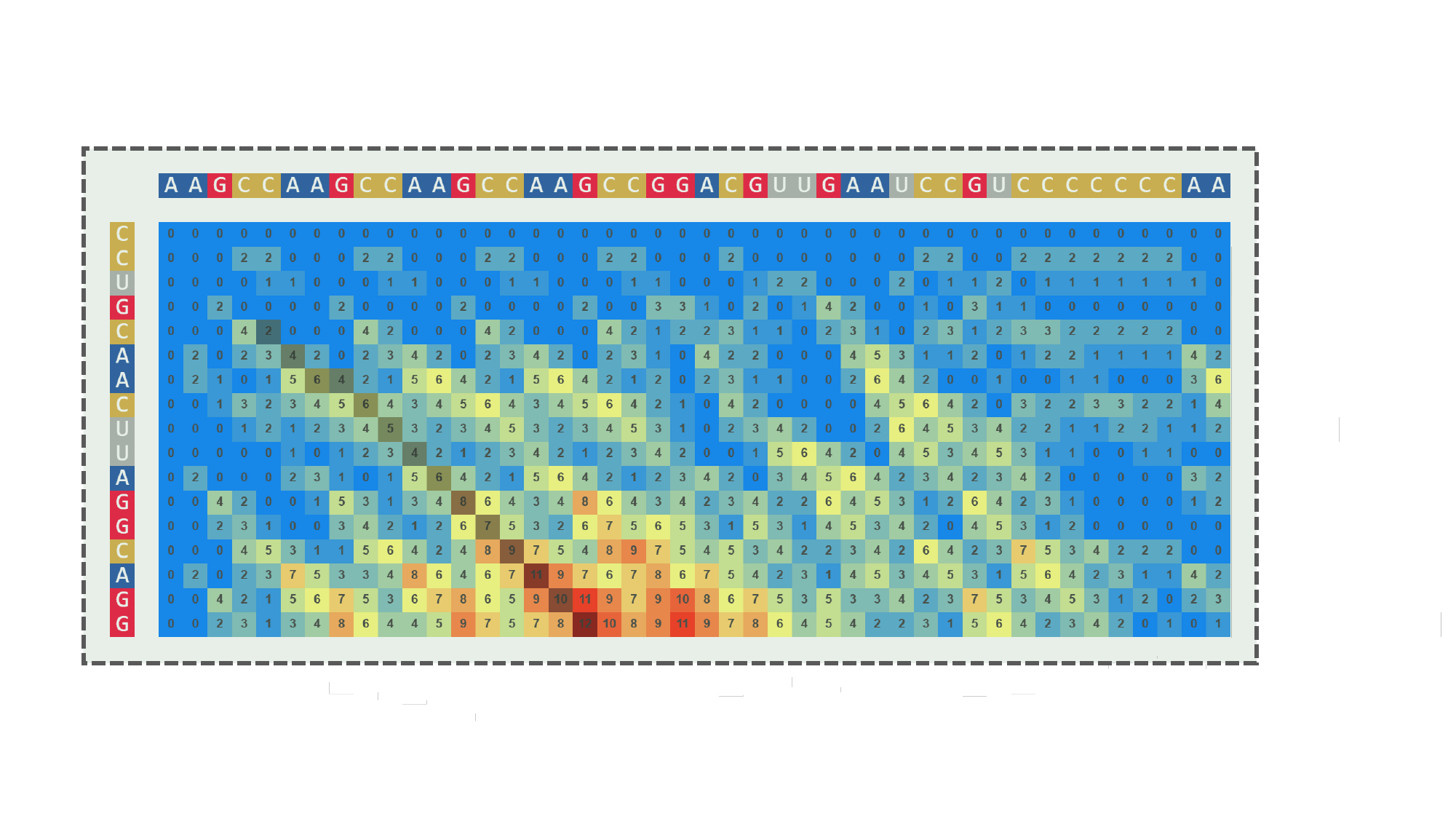

## Slide 6
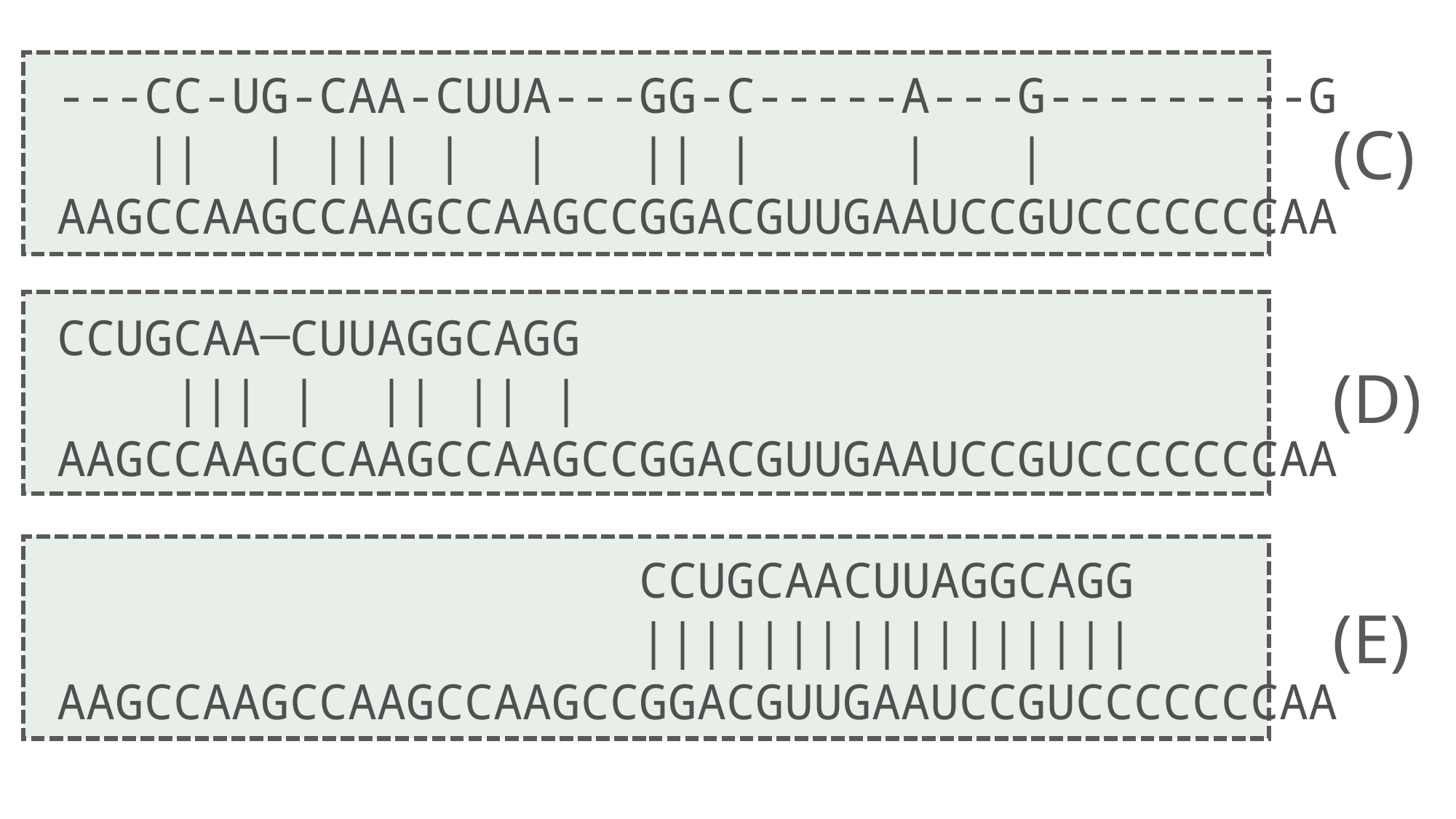

---CC-UG-CAA-CUUA---GG-C-----A---G---------G
   ||  | ||| |  |   || |     |   |
AAGCCAAGCCAAGCCAAGCCGGACGUUGAAUCCGUCCCCCCCAA
CCUGCAA─CUUAGGCAGG
    ||| |  || || |
AAGCCAAGCCAAGCCAAGCCGGACGUUGAAUCCGUCCCCCCCAA
 CCUGCAACUUAGGCAGG
                    |||||||||||||||||
AAGCCAAGCCAAGCCAAGCCGGACGUUGAAUCCGUCCCCCCCAA
(C)
(D)
(E)

## Slide 7
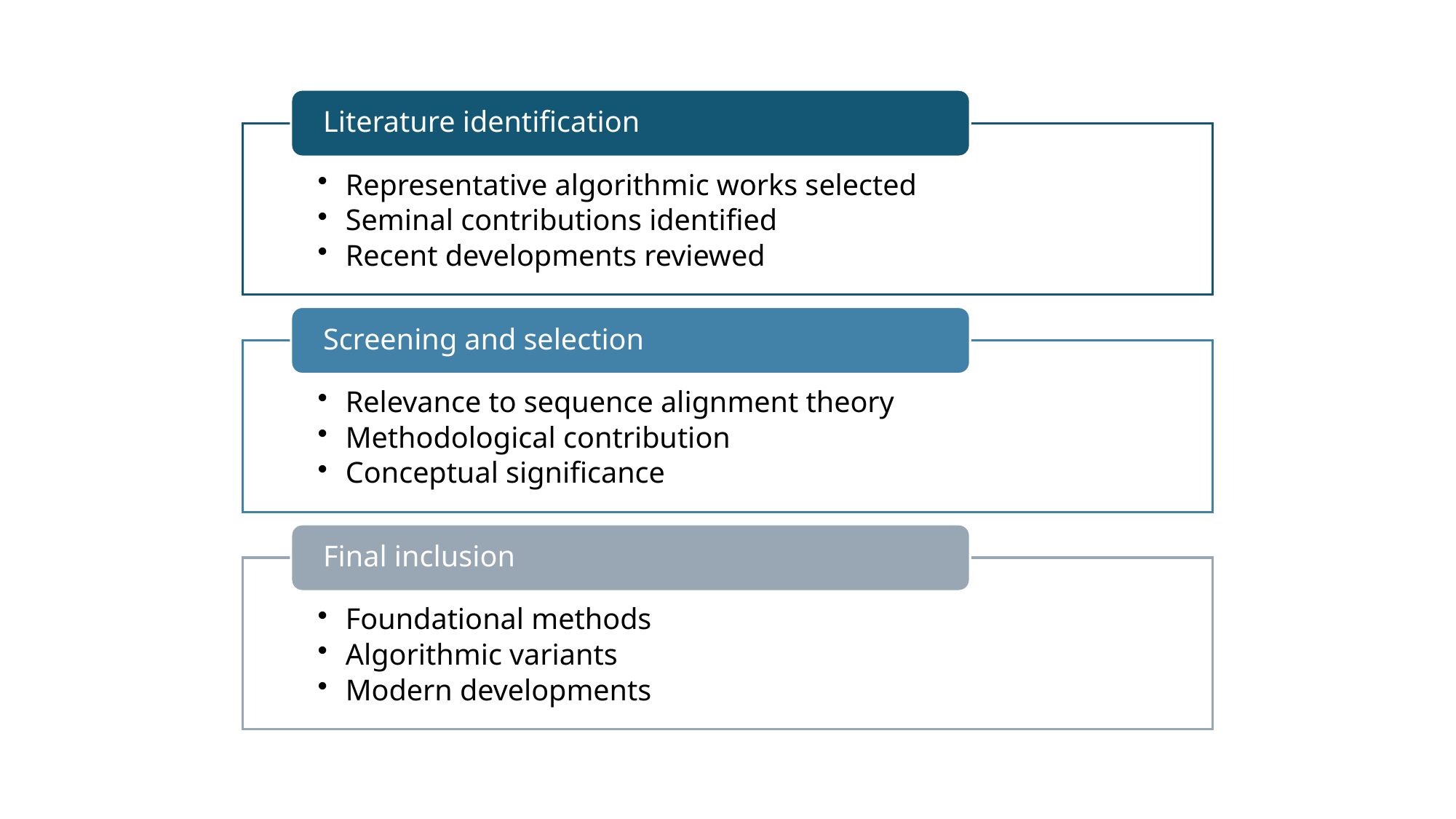

## Slide 8
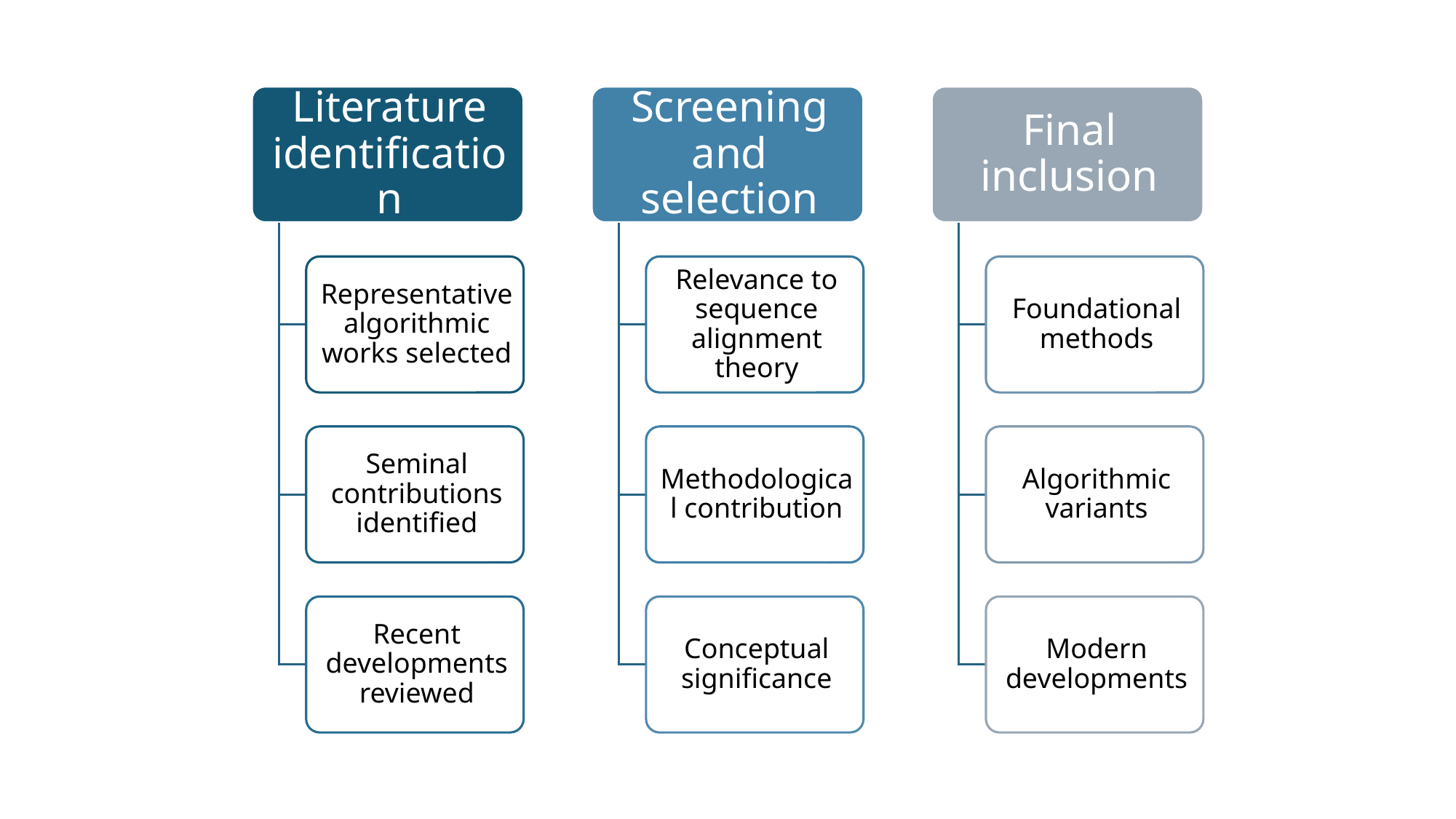

## Slide 9
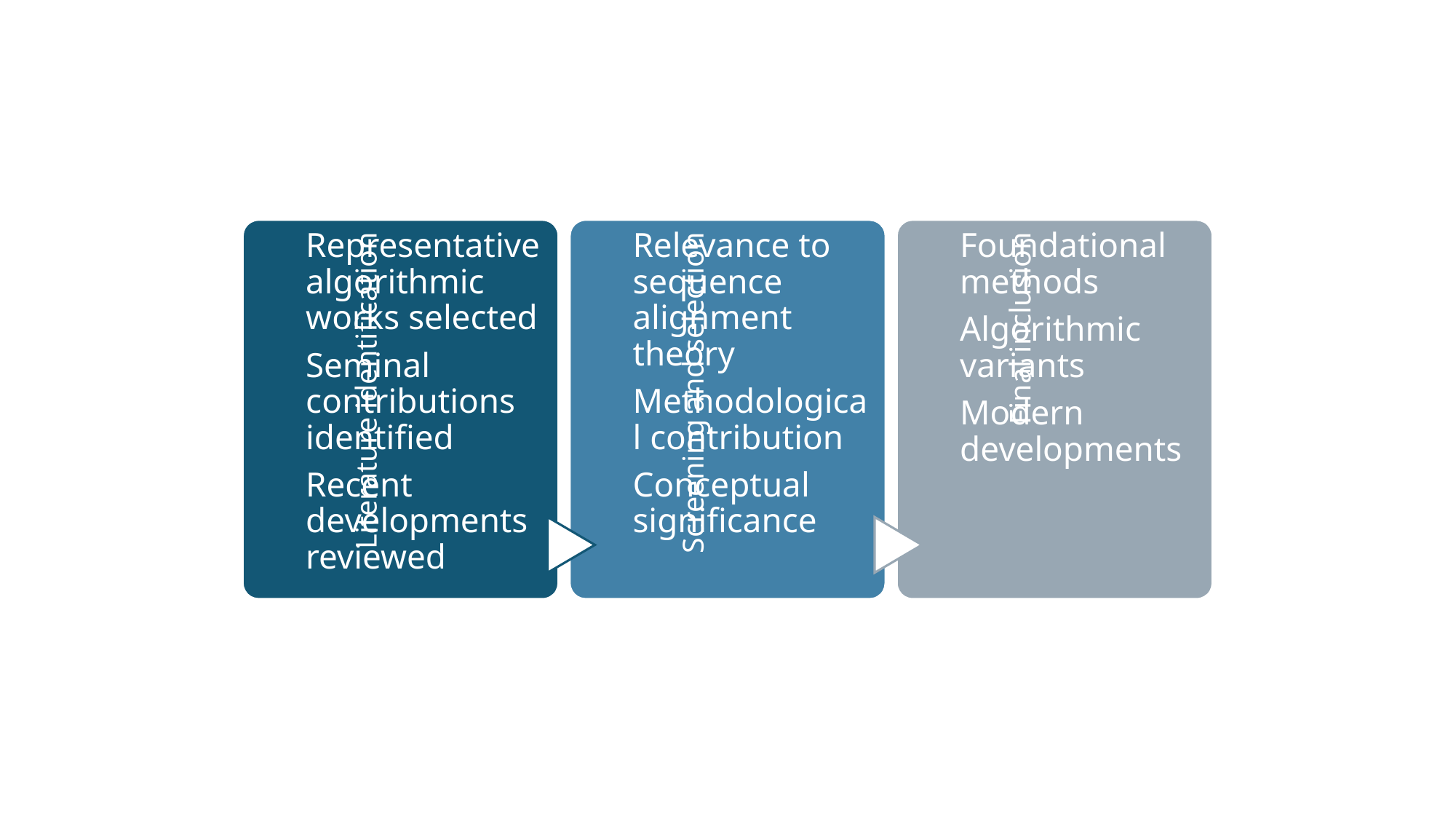

Supplement: Supplementary_material_bbag333 [file supplementary_material_bbag333.zip › design_bbag333.pptx]
