# Supplementary material for: The sequence alignment problem: boundary conditions as the unifying principle
Source: Brief Bioinform. 2026 Jun 21;27(3):bbag333. doi: 10.1093/bib/bbag333 (PMC13283437; doi:10.1093/bib/bbag333)
Supplement: Supplementary_material_bbag333 [file supplementary_material_bbag333.zip › Supplementary_Figure_S1_bbag333.docx]

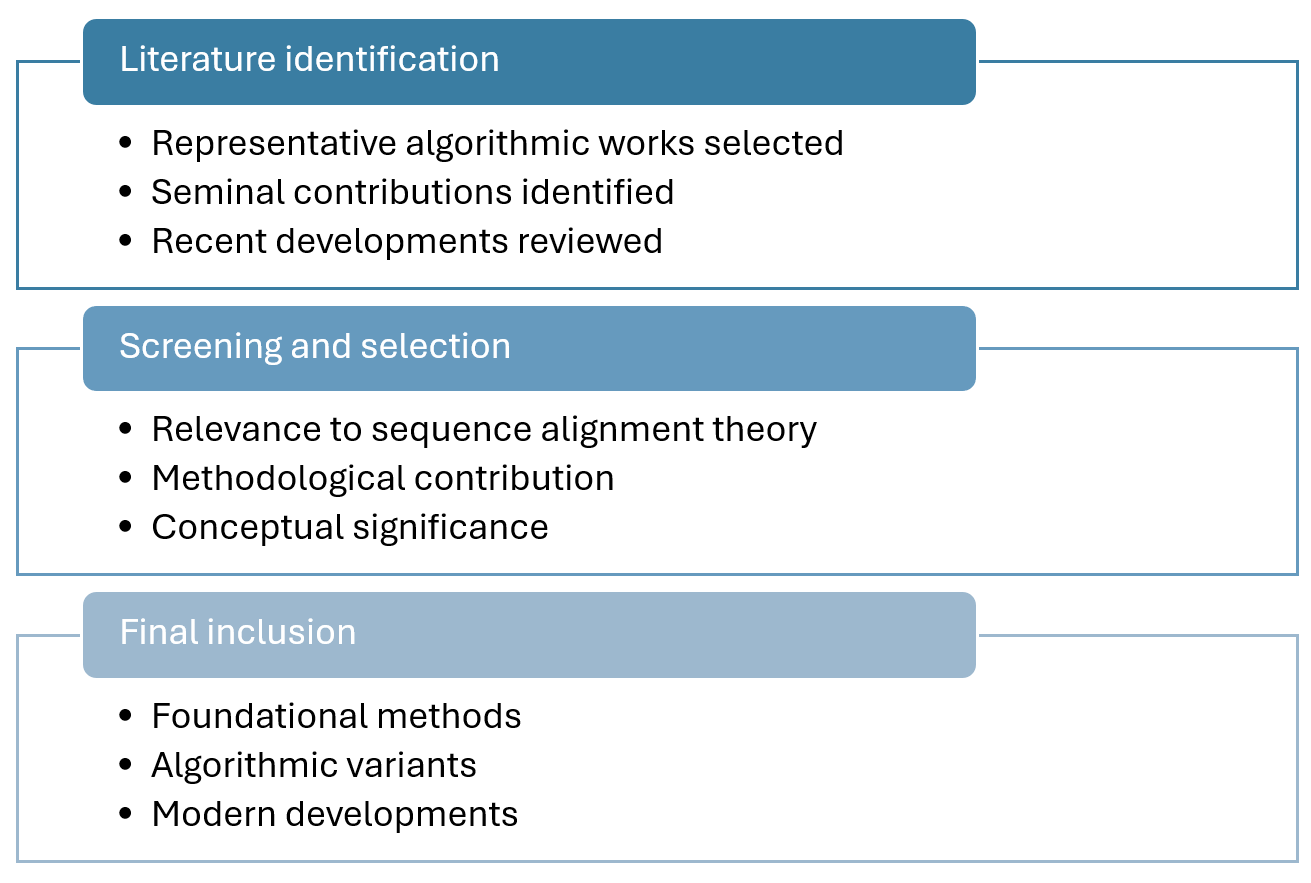


Supplementary Figure S1. Literature selection strategy for the present review. Schematic overview of the literature identification, screening, and selection process used to identify representative developments in sequence alignment. Studies were selected based on relevance to sequence alignment theory, methodological contribution, and conceptual significance, resulting in the inclusion of foundational methods, algorithmic variants, and modern developments.
