# Supplementary material for: The sequence alignment problem: boundary conditions as the unifying principle
Source: Brief Bioinform. 2026 Jun 21;27(3):bbag333. doi: 10.1093/bib/bbag333 (PMC13283437; doi:10.1093/bib/bbag333)
Supplement: Supplementary_material_bbag333 [file supplementary_material_bbag333.zip › Visualization_of_Sequence_Alignment_bbag333.html]

Bio


Run
Parameters
HeatMap
Alignment

Themes

Age of Empires
Alien
Dark
Dark Chem
European Union
Gentleman
Inferno
Military
Normal
USA
Red Alert

LocalStorage
Export PNG
⛶ Fullscreen

Copy Alignment

Plain Text
FASTA Format

✕ Exit Fullscreen


Copied to clipboard!

Run

░▒▓ Match

=

░▒▓ Mismatch

=

░▒▓ Gap

=

░▒▓ HEATMAP

░▒▓ MEAN & TRACE

░▒▓ TRANSPARENCY

░▒▓ Min

▓▒░

░▒▓ Mean

░▒▓ Middle

▓▒░

░▒▓ Density

░▒▓ Max

▓▒░

░▒▓ Path

░▒▓ Axes

▓▒░

░▒▓ Info

░▒▓ Cap

▓▒░

░▒▓ Cross

░▒▓ Letters

▓▒░

░▒▓ Numbers

░▒▓ HeatMap transparency:

░▒▓ HeatMap width:

░▒▓ HeatMap height:

░▒▓ Right chart width:

░▒▓ Bottom chart height:

░▒▓ Letters background:

in:

DNA

RNA

PROTEIN

NUMBER

░▒▓ CHR & COLOR

░▒▓ Significance

░▒▓
┏
▐
█▀
╔═
╔
[
⁅
⅀
Σ
ↁ
↦
⇀
⇒
⇝
⟾
🠶
🡆
🢚
≣
≫
⊂
⊏
⊑
⊪
⋉
⟥
⫍
⚑

▓▒░

Marker and color for Sq1

░▒▓
─
 
▄
■
▀
╦
┬
=

▓▒░

Gap and color of Sq1

░▒▓
█
|
║
■
╬
|
=

▓▒░

Match Sq1 vs Sq2

░▒▓
 
|
║
■
╬
░
▒
=

Mismatch Sq1 vs Sq2

░▒▓
─
 
▄
■
▀
╩
┴
=

▓▒░

Gap and color of Sq2

░▒▓
┗
▌
█▄
╚═
╚
[
⁅
⅀
Σ
ↁ
↦
⇀
⇒
⇝
⟾
🠶
🡆
🢚
≣
≫
⊂
⊏
⊑
⊪
⋉
⟥
⫍
⚑

▓▒░

Marker and color for Sq2

Hidde/Show DNA notation

Append names

Detect sequence type

Sequence match completeness

Letters/line:

70

Zoom in text:

22px

Line spacing:

2px

  
  

0%

---
